# Automating Agroecology: How to Design a Farming Robot Without a Monocultural Mindset?

**DOI:** 10.1007/s10806-021-09876-x

**Published:** 2022-01-22

**Authors:** Lenora Ditzler, Clemens Driessen

**Affiliations:** 1 Farming Systems Ecology Group, Wageningen University and Research, P.O. Box 430, 6700 AK Wageningen, The Netherlands; 2 Cultural Geography Group, Wageningen University and Research, Droevendaalsesteeg 3, 6708 PB Wageningen, The Netherlands

**Keywords:** Open-field agriculture, Mechanization, Pixel cropping, Crop diversification, Co-bot

## Abstract

Robots are widely expected—and pushed—to transform open-field agriculture, but these visions remain wedded to optimizing monocultural farming systems. Meanwhile there is little pull for automation from ecology-based, diversified farming realms. Noting this gap, we here explore the potential for robots to foster an agroecological approach to crop production. The research was situated in The Netherlands within the case of *pixel cropping*, a nascent farming method in which multiple food and service crops are planted together in diverse assemblages employing agroecological practices such as intercropping and biological pest control. Around this case we engaged with a variety of specialists in discussion groups, workshops, and design challenges to explore the potential of field robots to meet the multifaceted demands of highly diverse agroecological cropping systems. This generated a spectrum of imaginations for how automated tools might—or might not—be appropriately used, ranging from fully automated visions, to collaborative scenarios, to fully analogue prototypes. We found that automating agroecological cropping systems requires finding ways to imbue the ethos of agroecology into designed tools, thereby seeking to overcome tensions between production aims and other forms of social and ecological care. We conclude that a rethinking of automation is necessary for agroecological contexts: not as a blueprint for replacing humans, but making room for analogue and hybrid forms of agricultural work. These findings highlight a need for design processes which include a diversity of actors, involve iterative design cycles, and incorporate feedback between designers, practitioners, tools, and cropping systems.

## Introduction

A drive towards automation of both physical and cognitive work processes fuels increasingly ubiquitous applications of robots,^1^ for example in manufacturing, mobility, entertainment, health care, security, and food processing. In open-field farming^2^ too, robotization is being presented as inevitable (Blackmore et al., 2005; Harris, 2018). Commercial entities and research institutions are funneling tremendous resources into the development, testing, and production of robotic equipment for open-field settings, although these tools remain largely in research and development environments and have yet to gain traction among farmers the way applications in other agricultural realms have, such as automated milking systems (Bechar & Vigneault, 2016; Duckett et al., 2018). Nonetheless, in the wake of the COVID-19 pandemic it has been projected that investments into automated open-field farming technology will only accelerate (van der Boon, 2020).

Reading popular media coverage of advances in open-field agricultural automation gives a particular view of the way crop husbandry is being conceived, revealing the dominant monocultural approach—cultivating a sole crop in a given area—underpinning these developments. Recent headlines like those in *The Guardian* announce a particular direction for automation: “The rise of the robot farmer: We'll have space bots with lasers, killing plants” (Harris, 2018); or the latest, “Killer farm robot dispatches weeds with electric bolts” (Carrington, 2021). The dualisms evident in these titles (crop/weed, nature/(agri)culture) assume a particular approach to farming that is both precipitated and perpetuated by narrowly delimited measures of success.

The dominant narratives promoting robots in open-field agriculture anticipate that they will optimize the efficiency of agricultural tasks and inputs, thereby decreasing the need for human labor and the environmental impacts of monocultural, industrial agriculture in mechanized contexts (Bechar & Vigneault, 2016; Duncan et al., 2021). The potential for robots to increase the sustainability of industrial-scale cropping serves as a loud selling point (Duckett et al., 2018), as it is widely acknowledged that industrial monocultures contribute heavily to global environmental degradation and that alternatives are urgently needed (Campbell et al., 2017). Yet the expected gains in sustainability afforded by robotization are most often credited to the increase in precision with which they will apply agrochemicals or contain soil damage, and not to their capacity to facilitate agricultural practices that do not rely on environmentally problematic methods in the first place (Kuch et al., 2020; Miles, 2019).

Crop diversification has long been employed in farming systems as way to enhance ecological controls and spread risk and is gaining increasing attention in research and policy agendas as scientific findings point to its potential to mitigate the negative ecological impacts of monocultural production (Beillouin et al., 2021; Tamburini et al., 2020). Agroecological farming systems, which are grounded in ecological processes and knowledge and utilize various forms of crop diversification as a foundational practice (Francis et al., 2003), have been shown to provide agronomically, ecologically, and socially viable alternatives to the industrial monoculture model (Boeraeve et al., 2020; Juventia et al., 2021; van der Ploeg et al., 2019). Despite the fact that some form of mechanization (and possibly automation) will be necessary if diversified and agroecological approaches are to be translated and amplified in the industrialized contexts where they are most needed, technology makers have not yet focused intently on automation in such systems.

Following a well-documented trajectory of innovation in agriculture throughout which machines and farming systems have co-evolved (Magrini et al., 2019; Sassenrath et al., 2008), the majority of robots being developed for open-field arable and vegetable contexts are designed to function in monocultures and to fortify mutually reinforcing concerns of these systems: reducing labor inputs, increasing efficiency, and perfecting the uniformity of crop stands (Fountas et al., 2020). Key features of robots that could make them uniquely suited for applications in diversified settings—their potential to be light weight, modular or multifunctional, highly mobile, autonomous, and teachable—are not used to embrace heterogeneity, but are more often called in to further homogenize already monocultural production environments (Grieve et al., 2019). In addition to a lack of suitable tools being developed, there appears to be little pull from the agroecological, diversified farming realm to design automated farming machinery (Bellon Maurel & Huyghe, 2017), even though farmers who are interested in diversifying their cropping systems have cited a lack of appropriate tools as a substantial barrier (Morel et al., 2020). The question of what automation and robots for diversified agroecological farming could look like, be, or do remains largely unexplored.

In this paper we interrogate the push towards automation in monocultures, the lack of pull from the agroecological farming arena, and the associated critical debates on the agronomic potential as well as political ecologies that robots may imply, by exploring the potential for automating agroecology. We work with three guiding questions which address the concerns of automation from complementary angles:

- How could robots be made to embody the practices, interactions, and concomitant forms of care of farming agroecologically?
- What kind of (automated) machines would be considered suitable for the socio-political embedding of agroecological farming?
- How should the design processes needed to induce a change of sociotechnical practices towards amplified forms of diversified agriculture (which may include automation) be envisioned?

We explore these questions by first briefly reviewing the contextual background that led us—a farming systems scientist researching the potential of crop diversity experimentally and a cultural geographer curious whether robotization could foster ecological farming practices—to this inquiry. We then look at the guiding questions through the lens of an experimental effort at *pixel cropping* in The Netherlands, a nascent and complex intercropping method which offers a rich opportunity to understand the ways in which automation in novel agroecological, diversified cropping systems might differ from that in established industrial monocultures. Within the Dutch case, we held a series of interactive happenings which engaged practitioners from various disciplines around the challenge of how to make a pixel cropping robot. We integrated methods and learnings from the agricultural systems design literature and invited a range of systems actors in an effort to generate a diversity of approaches and responses to the same site-specific challenge. We analyze the ideas and challenges for automation that pixel cropping generated among participants in these happenings. We then explore the broader implications of the pixel cropping case, identifying key questions and possible ways forward for thinking about automation which could address the agronomic, ecological, socio-political, and design challenges of diversified, agroecological, open-field farming more generally.

## Historical and Conceptual Background

### Robots Pushed into Monocultural Fields

Agricultural innovation has for the last century been generally characterized by a trend towards bigger, heavier machines (Keller et al., 2019). This trend is evident in certain directions being pursued for automation in open field farming, manifested in tools such as driverless tractors and tractor-mounted ‘smart’ systems and sensors. These tools often explicitly aim to reduce or eliminate the need for human operators, an objective related to both social concerns and the agronomic co-evolution of monocultures and machines. In agriculture, technological innovation is often accelerated by societal transformations which reduce the availability of farm workers and increase the need for labor efficiency (e.g. war, the abolition of slavery, changes in immigration policy, pandemics), resulting in advances in mechanization which bring particular political ecologies into being that foster large scale monocultures with concomitant environmental impacts and social transformations (Rasmussen, 1982; Vandermeer, 1986). When sole machines replace teams of human workers, the time required to perform cultivation tasks on a per hectare basis is dramatically reduced,^3^ allowing farmers to cultivate larger tracts of land by transforming “walking tasks” into “driving work” (Schmitz & Moss, 2015). Farmers who invest in expensive and large machines often then face a need to specialize and upscale in order to maximize resource use efficiency, compete in economies of scale,^4^ and make their investments worthwhile (Scott, 1999). This feedback process thus produces a particular socio-political dynamic of modernization, combining chemical and other inputs with capital requirements and dependence on large agribusiness conglomerates, while rural and migrant communities disappear due to reduced or only seasonal, transient labor needs (Carolan, 2019; Friedmann, 1999; Rotz et al., 2019; Schmitz & Moss, 2015).

A key factor in the drive towards large machines, automated or not, is the need to maximize the uniformity of a crop stand. Harvesting maize with a combine, for example, is only effective if all the plants in the crop stand are the same height, produce ears of the same shape, and reach maturity at the same time. Additionally, the more of the same type of plant there are to harvest in a given area, the higher the potential productivity per unit of land or labor. As such, plant breeding programs and seed suppliers, in combination with chemical inputs, have focused heavily on growth characteristics (e.g., height, crowding tolerance) that are relevant for optimizing monocultural, machine-managed cropping systems (Bourke et al., 2021; Kloppenburg, 2005). Together, these factors create a mutually reinforcing feedback loop whereby cropping systems are engineered to accommodate large machines and large machines are designed to manage engineered cropping systems (Scott, 1999; Miles, 2019; Lowenberg‐DeBoer et al*.*, 2021).

The interlinked drivers of efficiency and uniformity lead towards the final challenge of total automation: how to do arable farming without needing human workers in the field. In addition to large driverless tractors, this challenge is being pursued through the development of small autonomous robots^5^ (Relf-Eckstein et al., 2019). These go against the bigger—heavier mechanization trend, but most often still assume a monocultural approach to crop husbandry despite their potential to determine a different relationship between farming systems and machines. Open-field robotic applications come predominantly out of the precision agriculture program, which focuses on the use of technology and data to enhance resource use efficiency and maximize production through targeted, plant-level care within monocultural systems (Lowenberg-DeBoer & Erickson, 2019). Here, the robotization of agricultural tasks is touted as a solution to problems of within-field variability, imprecise and overabundant agro-chemical application, soil compaction, and the high cost and low availability of farm labor (Blackmore et al., 2005; Kuch et al., 2020; Murray, 2018). Applications often involve (multiple) autonomous ground units receiving and processing information from various forms of sensors (Fountas et al., 2020). Through abundant data collection, it is expected that these robots will help farmers achieve a greater measure of uniformity in their cropping systems by identifying, diagnosing, and treating plant-level heterogeneities in the field environment in a resource-efficient manner (Pedersen et al., 2006). Widespread adoption of this autonomous farming equipment would mean the shifting of manual labor demands off human farm laborers and onto robots, potentially aggravating the often racialized and gendered marginalization of agricultural laborers in some contexts (Marinoudi et al., 2019; Rotz et al., 2019; Sparrow & Howard, 2020). With advances in artificial intelligence (AI), the cognitive human demand may also eventually be made optional, calling into question what it means to be a (‘good’) farmer (Burton, 2004; Driessen & Heutinck, 2014).

### Agroecological Pull?

When considered as a potential systems innovation for accelerating sustainability transitions in agriculture, the lack of attention given to tools that enable agrobiodiversity demonstrates a lock-in within the monocultural system. The dominant socio-technical regime is robust, favoring developments that fit within a monoculture approach and positioning diversified cropping systems in a niche outside the boundaries of the prevailing innovation landscape (Geels, 2002; Morel et al., 2020). In part, this lock-in is influenced by the fact that heterogenous field designs pose many more agronomic and technical challenges than monocultures: it is simply more difficult to mechanize or automate the management of a polyculture compared to a sole crop (Fountas et al., 2020). Additionally, an apparent lack of consensus as to what role automation should play in the transition towards a more sustainable agriculture may be impeding advances in automation for diversified cropping systems (Bellon Maurel & Huyghe, 2017; Herrero et al., 2021; Shepon et al., 2018).

Within agroecology, there is an emphasis on social empowerment, equity, and sovereignty (Altieri & Toledo, 2011), in addition to a strong agronomic tradition of crop diversification (Wezel et al., 2014). However, there is little academic focus on the question of how to make physical tools for agroecological practices (cf. Salembier et al., 2020). Even in a recent comprehensive overview of the research needed to apply agroecology specifically in large-scale farming contexts, written particularly from an agronomic perspective, mechanization challenges were not addressed (Tittonell et al., 2020). Although often correlated justly with specialization, upscaling, capital intensity, and other tropes of the monocultural paradigm (van der Ploeg, 2021), there appears to be no fundamental reason why automated tools could not be designed to progress agroecological aims. In fact, research has shown that farmers themselves are not necessarily the ones worried about the incompatibility of technology and ‘alternative’ farming methods (van Hulst et al., 2020). That said, recent syntheses also show that adoption of ‘smart’ farming technologies is lower among ‘unconventional’ farmers than among ‘conventional’ (i.e. intensive, high-input) farmers (Bronson, 2019).

Some see the lack of attention put on automation as a barrier to the amplification of agroecology (e.g. Bellon Maurel & Huyghe, 2017), believing that where labor is a limiting factor, automation offers the possibility of implementing agroecological practices in new contexts and at broader scales. In painting a picture of an automated ecological farming utopia, Daum (2021) imagines that fleets of robots working 24/7 will enable farmers to adopt agroecological farming methods where high labor demands would otherwise be a constraint. For others, particularly in small-scale systems and less mechanized contexts, the manual labor demands of ‘doing’ agroecology are rather regarded as opportunities to foster meaningful livelihoods and community involvement, connecting humans both to the land and to each other (Nicholls & Altieri, 2018; Timmermann & Félix, 2015). From this perspective, a fear is that tools such as driverless tractors or autonomous robots may undermine the intrinsic value of being a farmer, displace workers, or lock farmers into disadvantageous power asymmetries (Carolan, 2019).

A hesitant stance towards technology fits into a long history of critiques questioning the societal effects of mechanization in agriculture (Fitzgerald, 1991; Vandermeer, 1986), and contemporary critical perspectives on the potential social and ethical concerns raised by automated farming technologies are abundant (e.g. Klerkx & Rose, 2020; Rose & Chilvers, 2018; Ryan et al., 2021; Sparrow & Howard, 2020; van der Burg et al., 2019). Emerging key issues relate to data ownership, deskilling, exclusionary power dynamics, and shifting farmer identities (Klerkx et al., 2019). In conversation with these assessments are abundant calls for participatory and reflexive design processes in the development of responsible innovations for sustainable farming (e.g., Berthet et al., 2016; Cerf et al., 2012; Eastwood et al., 2019; Elzen & Bos, 2019; Jakku & Thorburn, 2010; Lacombe et al., 2018; Pissonnier et al., 2019; Prost, 2021; Rossing et al., 2021). Such ‘user-centered’ design methods are often seen as a way to incorporate socio-political concerns into design specifications, and frequently take a systems perspective at the farm, value chain, or regional scale. Within design discussions, however, the challenge of translating the unique agronomic, ecological, and social demands of diversified cropping systems into designs for agricultural implements (let alone automated machines) is often peripheral and secondary to the design of the farming system itself (see e.g. Prost et al., 2017; cf. Salembier et al., 2020). On the other side there is an abundance of literature describing advances in open-field agricultural robotics (e.g. Bechar & Vigneault, 2017; Fountas et al., 2020; Kootstra et al., 2021; Mahmud et al., 2020; Oberti & Shapiro, 2016), but studies combining design processes geared towards diversified agriculture settings with automated tool specifications are noticeably lacking (Rose et al., 2021).

### The Prospect of Pixel Cropping

**Fig. 1:**
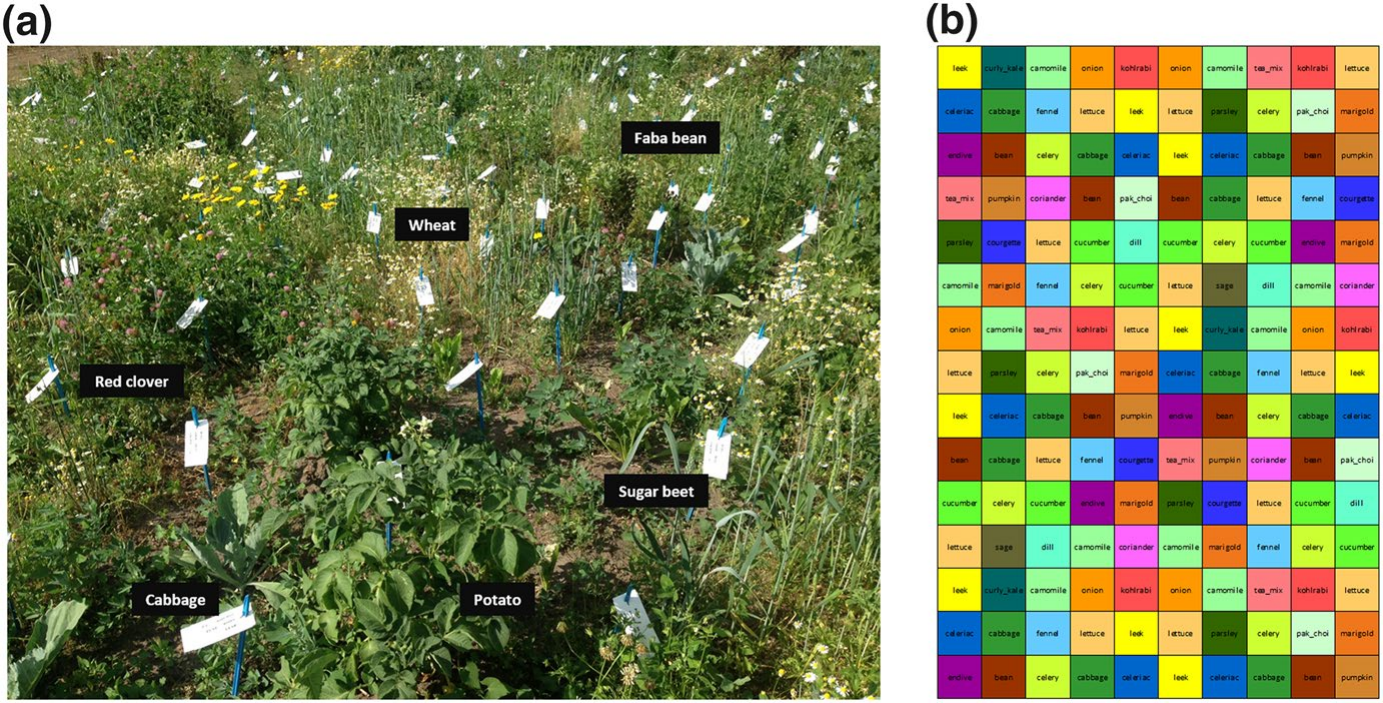
A pixel cropping plot at the Wageningen University field trial on the Droevendaal Organic Experimental and Training Farm, NL in which six crops are planted in 0.5 m × 0.5 m pixels in plots of 9 m × 12 m (**a**); and a subset of the 2020 pixel field planting plan at the Lochem, NL trial in which 30 crops are planted in 1.5 m × 1.5 m pixels in a 1 ha field (each color represents a different crop) (**b**)

Pixel cropping (sometimes referred to as *pixel farming*) occupies a unique space of being both grounded in agroecology and dependent on technology. It is an open-field farming method in which many different food and service crops are planted together in diverse assemblages made up of small (0.25–2.25 m^2^) crop patches (‘pixels’) arranged in a grid (Fig. 1) (Ditzler & van Apeldoorn, 2018). It employs multiple agroecological techniques that increase temporal, spatial, and genetic in-field diversity (rotation, intercropping, crop mixtures), conserve soil (continuous cover, green manures), and facilitate natural pest control (habitat and resource contiguity and continuity). Pixel cropping as we describe it here was developed recently in The Netherlands following a ‘Cartesian’ orthogonal logic (Ditzler, 2020), although it draws heavily on the principles of established agroecological methods that leverage diversity, including companion planting and indigenous intercropping practices such as the milpa or Three Sisters (Lopez-Ridaura et al., 2021; Rodríguez-Robayo et al., 2020).

Although a pixel plot is organized in a grid (for ease of management and scientific study) which resembles the pixelated field maps used to collect and present data in precision agriculture, the concepts underlying pixel cropping are not about precision. Rather, the method aims to maximize the structural diversity of the arable field, and in doing so create a production system that functions as a healthy ecosystem. Algorithmic inputs may be utilized in the design of a pixel field (e.g., following rules for which crop—neighbor combinations to encourage or avoid, or the outputs of detailed soil mapping), so in this way pixel cropping does not exclude the digitalization underpinning precision agriculture, but homogenization is not an aim. After a pixel plot is sown, what develops may appear wild and unruly compared to conventional agriculture. The high-resolution of individual pixels creates an assemblage of ecological interactions, generating multiple dimensions of habitat, resource, and functional diversity which occasion emergent properties beyond production—what Tsing (2015, p. 23–24) refers to as the “multiple temporal rhythms” and “patterns of unintentional coordination”—that can be understood as agro-ecosystem services.

There is strong scientific evidence that high resolution in-field diversity and small field sizes will indeed produce good yields *and* abundant ecosystem services (Fahrig et al., 2015; Sirami et al., 2019; van Oort et al., 2020), so pixel cropping is anticipated to be a promising approach for addressing emerging production and environmental sustainability aims such as those outlined in recent Dutch and European policy (EC, 2020; Schouten, 2019). However, pixel cropping is not yet employed outside of a limited research-oriented context, so the broader scope of the method for meeting these aims—as well as its profitability and global applicability—have not yet been explored. At the time of writing only three pixel cropping trials were known to the authors, all in The Netherlands. In part, pixel cropping’s limited uptake has to do with the management demands posed by its planned and emergent complexity. Labor presents a major challenge in mainstreaming or upscaling the method, particularly in places where intensively mechanized farming is the norm, as in The Netherlands. Due to the small size of individual crop patches and the heterogeneity of the overall field layout, conventional machines cannot be used to conduct tasks such as sowing, weeding, or harvesting, meaning that currently all labor must be done by hand. There is consensus among those who have worked in pixel cropping trials that implementing the system at viable scales will require some form of mechanization, and potentially automation. However, no technologies that could work in such high-resolution and large-scale intercropping systems are yet on the market. The lack of established tools, in combination with its future-oriented outlook, positions pixel cropping as a unique case for imagining non-monocultural possibilities for automation without the influence of an already saturated solution space and within a still exploratory phase of experimentation.

## Empirical Approach

The discussion groups, workshops, and design challenges that this paper is based on are embedded in the Ph.D. research project of the first author. The project was approved by the governing graduate school at the start of the Ph.D., and in designing and conducting the research for this paper we followed the Netherlands Code of Conduct for Research Integrity as formalized by our institutional Social Sciences Ethics Committee. The committee provides a checklist which researchers should use to determine if their research requires an ex-ante ethics review; according to these guidelines, we determined that our study met the ethical criteria and therefore did not necessitate review by the ethics committee.

The research was conducted between 2018 and 2021 in and around an existing pixel cropping field trial at the Droevendaal Organic Experimental and Training Farm located on the Wageningen University & Research (WUR) campus in Wageningen, and at an on-farm pixel cropping trial in Lochem, both in The Netherlands. Both trials are embedded within ongoing, long-term agroecological studies monitored by WUR researchers. The trial in Wageningen started in 2018 and consists of two experimental pixel cropping plots (each 9 m × 12 m) managed for scientific purposes (Fig. 1a) (see Juventia et al., (2021) for details of the experimental design). The Lochem trial was initiated in spring 2020 and is conducted on a working organic farm where the resident farmer uses the 1 ha pixel field (Fig. 1b) for both research and commercial purposes.

Centered around these two trials we explored how a diversity of practitioners from different disciplines might approach the same site-specific challenge. Drawing on methods commonly employed in agroecological research (action research (Lieblein et al., 2012), Kolb’s learning cycle (Kolb, 1984), the DEED cycle (Giller et al., 2011)), we designed a series of interactive happenings which progressed in topic and depth over the research period, moving from broadly positioning the question of automation within the frame of general agroecological concerns towards envisioning specific applications for robotics in the Dutch pixel cropping context. In view of the explorative character of this study, we approached the research design and analysis as an iterative process (Locke et al., 2020) in which we adapted the set-up and focus of the happenings and composition of the participants involved by drawing on observations that emerged in the process.

At the Wageningen site, we employed various interactive formats (a group discussion, a World Café-style workshop, a design challenge, and a consultancy project) to engage students, researchers, and practitioners from a range of fields in place-based efforts to explore the potential for robots to facilitate pixel cropping (Table 1). Most of the participants were employees and students from relevant departments of WUR, representing a range of nationalities and disciplinary backgrounds. The workshop participants also included professional designers, farmers, and independent agro-tech developers from outside the university, and the design challenge involved students from the Design Academy Eindhoven (DAE), again with international backgrounds. Both authors took extensive notes during the Wageningen meetings on the ideas that were put forward in the discussions as they occurred, in the larger gatherings with the help of a research assistant. We also archived the written and drawn outputs that were produced by participants.

**Table 1:** Overview of research happenings conducted from 2018 to 2020 in the context of the Wageningen University pixel cropping field trials in The Netherlands, presented in chronological order

Setting	Participants	Format	Guiding questions
Group discussion	5 agroecology-focused farming systems researchers working at WUR	Participants were posed with two open-ended questions and asked to freely discuss. Conversation topics were annotated on flipcharts	1. What is agroecology and how do farmers implement it? 2. What would a farming robot need to do to be in line with these principles and practices?
World Café workshop	20 robotics experts, ecologists, agronomists, farmers, and designers (both from WUR and from outside the organization)	Participants were given a tour of the Wageningen pixel cropping field experiment and presented with a list of issues and desires for a diversified farming systems robot synthesized from the previous discussion group. They then rotated through mixed groups where in each session they were asked to imagine different elements of appropriate forms of automation for the pixel cropping context, culminating in a design session where the elements were integrated and presented in drawings	1. What are the ecological, agronomic, and social requirements of a pixel farming robot? 2. How could/should these functions be integrated into an actualized design?
Challenge-based design course	20^a^ second-year bachelor design students from DAE	Students were asked to respond to the idea of robots as an approach to dealing with the manual labor challenges of pixel cropping. They were first given a general introduction to the principles of pixel cropping and a tour of the Wageningen field experiment, where they interviewed the field staff responsible for conducting daily crop management tasks. They then worked independently on their design projects for 8 weeks. Prototypes were presented in a studio critique setting	1. How can the manual labor challenges of pixel cropping be solved?
Consultancy project	6 WUR MSc students	A team was commissioned to explore the outlook and design of robots for pixel cropping. Students were asked to identify the agronomic, ecological, and labor needs of pixel cropping and to design a prototype pixel cropping robot based on their findings. The team’s designs were presented in an oral presentation and written report	1. What are the agronomic and ecological demands of pixel cropping? 2. What is the state of the art in agricultural technology to mechanize these demands? 3. What are the most promising options for integrating these functions?

^a^The course began with 20 students split into three teams. Only one team (6 students) followed the pixel cropping labor challenge through to the end; it is that team whose work we report on in the results

In parallel to these orchestrated happenings, we also followed the progress of the self-identified agroecological farmer’s first season of doing pixel cropping at the Lochem site. The farmer was collaborating with a robotics developer to test a prototype weeding robot, and we paid particular attention to the farmer’s experiences with the robot and his widening effort to make pixel cropping work beyond the functioning of the robot. We conducted multiple unstructured interviews with the farmer before the growing season, and one semi-structured interview each during and after the growing season. The first author also visited the farm on several occasions, once to watch a demonstration of the robot testing its functions. We recorded the semi-structured interviews with the farmer, took notes during unstructured interviews and after field visits, and made images during field visits.

After each happening, interview, and field visit, we reviewed the archived material and inventoried the emergent themes in relation to our three guiding questions ("Introduction" section), which we coded as *work*, *community*, and *design*. We then used these data as input for guiding the next happening, with the aim to broaden the imaginations being represented while maintaining a relevant focus. For example: the themes we inventoried from the discussion group were used to select the range of participants and write the prompts for the World Café workshop; the outcomes of the World Café workshop led us to develop a design challenge with students not already involved with agricultural robotics; our observations of the farmer’s unanticipated challenges with the robot led us to reorient our focus around the broader context of his efforts at making the pixel farm work in practice; and our discussions with the farmer led us to ensure that an opportunity to talk with and observe someone doing pixel cropping labor was part of the student projects. Throughout this iterative process of interaction, coding, and analysis, we sought to identify variation between and within the groups that responded to or diverged from common discourses and assumptions (also ours) of what agricultural robots can or should do. To ensure that we stayed up to date with these discourses, we periodically attended agricultural robotics conferences, demonstrations, field days, and promotional events within and outside WUR; we did not collect data during these activities, but they were pivotal in helping us gain a broader view on developments in agricultural robotics in both research and commercial realms.

## Results: How to Make a Pixel Cropping Robot?

### Agroecology As an Ethos

Our inquiry began by seeking to position the specific question of automating pixel cropping within the broader framing of diversified agriculture via agroecology, asking a group of agroecology-focused farming systems researchers to define agroecology. During this discussion, the participants focused primarily on the conceptual aspects of agroecology, rather than the science or practice components that have been defined by other scholars (see Wezel et al., 2009). Several used language to describe agroecology as a stance towards farming that believes nature should be worked with rather than against. One participant explained, farming agroecologically means using a localized approach in which you start with “what the ecosystem offers” and seek to “understand the function of each inhabitant of the ecosystem” and then design farming interventions based on nature’s “template,” rather than the other way around. Another participant, who was from Southeast Asia, described expressions of agroecology in her community as being linked to a religious edict prohibiting the harm of nature. Participants returned often to the theme of connectedness, in reference to agroecology being about embracing and intensifying connections—e.g., between plants and earthworms, between farmers and their soils, or between farms and communities. How participants envisioned such relationships was also characterized by a localized contextualization; despite their different cultural and geographical backgrounds, all participants referred to agroecology as being necessarily adapted to local circumstances and belief systems, resulting in different connections to be emphasized in different contexts.

The way the participants envisaged agroecology appeared to imply that to practice agroecology is to invest in the connections, both ecological and social, that root farming and food systems in local ecologies and cultures. Interestingly, we witnessed no discussion about how these values and approaches could or should be implemented in practice when participants defined agroecology. The group in fact did not talk about work at all, never addressing what it looks like to physically execute agroecological practices. More generally, discussions of the importance of farming in a way that is attuned to ecologies and the temporalities of soils and other aspects of agroecosystems do not necessarily link to the challenges of everyday embodied practice. In these discussions the various aspects of an approach such as agroecology can be better understood as describing an ethos,^6^ within which knowledge is a key theme, but reference to forms of mechanization (let alone automation) are not often made (Puig de la Bellacasa, 2015; Sanderson Bellamy & Ioris, 2017). The implications of this first discussion group suggest that an agroecological approach to automation should consider the ecological relationships and cultures motivating the material practice as equally as relevant as technical specifications, generating the question of how to inscribe an ethos in agricultural machinery.

### Neighbors, Ducks, or Robots?

In the second part of the conversation with the farming systems researchers, we centered the question of possible synergies or tensions between automation and the previously described aims of agroecology. Here we noted an important difference in the focus of the discussion: when we asked about automation, participants brought up broader issues around the work of doing agroecology, which we could map onto longer-standing debates about the political ecologies of agricultural mechanization as discussed in the "Robots Pushed into Monocultural Fields" section. One participant (a farmer himself) reflected that during a tactile task like manual weeding, a farmer is “not just weeding” but also observing the crop, the soil, and the environment, the manual labor thus affording something more than could be gained if he did the task with a machine. On the other hand, he noted, “after three hectares, [hand] weeding is not so ‘Zen’ anymore.” This tension between the desirable sensory—and even meditative—experience and the drudgery of doing farm labor was echoed in a subsequent discussion about the potential advantage of automation as a tool to open up time, in which participants simultaneously championed the embodied knowledge that a farmer can accrue through physical labor and acknowledged that eliminating such labor might free the mind to explore opportunities for system “redesign” (Meynard et al., 2012).

When we asked participants specifically what a robot would need to do to fit into their definition of agroecology, they focused on the role the robot might assume in a farming community, which differed between their working contexts and the social norms and investment capacities available there. The participant from Southeast Asia explained that in her community many people need work, and she would rather hire a neighbor to weed her field than employ a robot. Conversely, a participant from a Nordic country noted that he would rather avoid talking to his neighbors and therefore would welcome a robot. When asked about tasks that may not be suited to human hands, such as controlling pests in a rice paddy, the Southeast Asian participant replied that robots were still not needed because “we have ducks for that.” The question of whether a robot would be regarded in particular contexts as either desirable (reducing the drudgery of labor, affording time for system redesign) or undesirable (displacing valued neighbors or ducks) could be seen as a contemporary iteration of discussions around the definition of progress in agriculture, flagging the continued relevance of long-standing calls to consider the localized social, cultural, political, economic, and ecological conditions that surround proposed technological change as well as the non-economic reasons farmers may have for pursuing particular technologies (Fitzgerald, 1991; Harwood, 2013; Sparrow & Howard, 2020; Vandermeer, 1986).

### What Should be Automated?

After framing the concepts and issues underpinning the general question of automation in agroecological farming systems, we moved into the happenings targeted specifically at pixel cropping (the World Café workshop at Wageningen, the challenge-based design course with students from the design school, and the consultancy project with WUR students). Although different, the entry points of both the World Café workshop participants and the two student groups seemed to reveal an underlying assumption that all needs of the cropping system—whether cultivated or associated—should be considered as demands that the hypothetical robot might be asked to control. As such, the approaches revealed the range of technical and conceptual considerations that would need to be accounted for in order to achieve full automation of a pixel cropping system.

The participants in the World Café workshop were most familiar with the ecological concepts behind pixel cropping and the agronomic practices employed in it, and began with a broad, holistic approach focusing on factors central to understanding and maintaining agroecological cycles and feedbacks at the foundation of the pixel cropping logic (defining system boundaries, identifying performance indicators, optimizing ecological interactions). Here participants emphasized that automating a pixel field requires a whole-system approach and a complete understanding of all the complex interactions involved. Both student groups were new to pixel cropping (the design school students had generally no experience with agriculture at all) and sought to first understand more concretely what the method entailed in practice, and then created inventories describing the production cycle of all crops in the Wageningen experiment trial and the cultivation tasks required at each phase that a robot would need to assume control of.

### Picking, Shaking, Cutting, De-leafing: Defining Functions

The three happenings followed similar trajectories in their next steps, moving from defining system boundaries and demands into discussions of how automated tools could technically meet those demands**.** Participants approached the task of defining tool functions from two general angles: starting with the command center in a top-down approach, and from individual tasks from the bottom up. These appeared as complementary and equally necessary elements of the pixel cropping robot design process.

In the World Café workshop, several groups discussed the decision-making functionalities underpinning the actuation of robotic equipment. Here, participants explored the possibilities to use AI, modelling, and various forms of sensing to enable autonomous decision making, leading into questions of what role the farmer would have in relation to AI-driven systems and how much autonomy should be afforded to robotic tools. A farmer in one of these design groups (the same farmer managing the Lochem trial) expressed that no matter how many sensors robots might be equipped with, he would prefer to make the ultimate decisions himself about what functions a tool performs, stating that “the farmer *is* the sensor.” Robotics engineers in the same group related to the farmers’ preference as a need to build into the robot “the power to overrule”—that is, the option for a human operator to override the machine’s autonomy at any moment. In the agricultural robotics literature this functionality is often referenced in regards to safety, cited as necessary for situations where the robot might be in danger of harming a person or itself (Vasconez et al., 2019). However, in the workshop, the connotations of the “power to overrule” took a different slant in the context of the previously defined system demand for holistic knowledge of agroecological interactions, of which it was acknowledged that the farmer would need or want to contribute to.

**Fig. 2:**
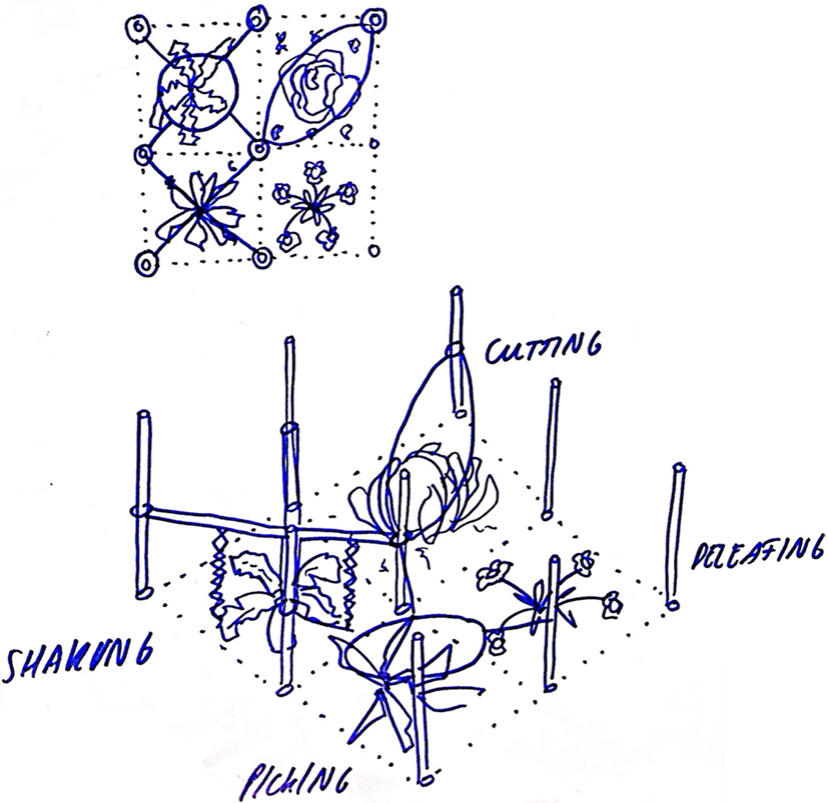
Picking, shaking, cutting, de-leafing: multiple ways to achieve the same task, illustrated by participants in the pixel cropping robotics design workshop in Wageningen, 2019

**Fig. 3:**
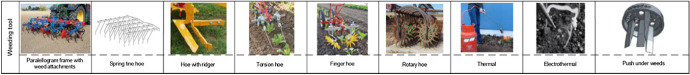
Morphological chart created by a WUR student consultancy team for addressing the mechanical weeding function of a pixel cropping robot, 2020

Other groups at the World Café workshop, as well as the student consultancy team, took a more grounded approach to matching technical possibilities with the system demands previously identified. For example, one workshop group discussing the challenge of harvesting crops in pixel plots diagramed four possible ways a crop could be mechanically harvested (Fig. 2). Similarly, the consultancy team created morphological charts, a method employed in the Reflexive Interactive Design methodology (Elzen & Bos, 2019), to systematically identify tools that could execute each cultivation task (Fig. 3). Interestingly, the consultancy team decided at this stage that automating the whole production cycle was too elaborate to solve with a single tool and chose instead to focus only on seeding and weeding cereals, the tasks they identified as the most labor-intensive at the pixel cropping field trial. The design school team came to a very similar conclusion, determining that addressing the whole production cycle with a single tool was unrealistic. Having conducted an extensive interview with the Wageningen pixel cropping field technician and carefully observing her movements as she performed various tasks in the field, the design students identified seeding cereals as the most arduous of the field operations and decided to focus on specifying the requirements for a tool that could alleviate this particular drudgery.

### Integration: High-Tech, Low-Tech?

**Fig. 4:**
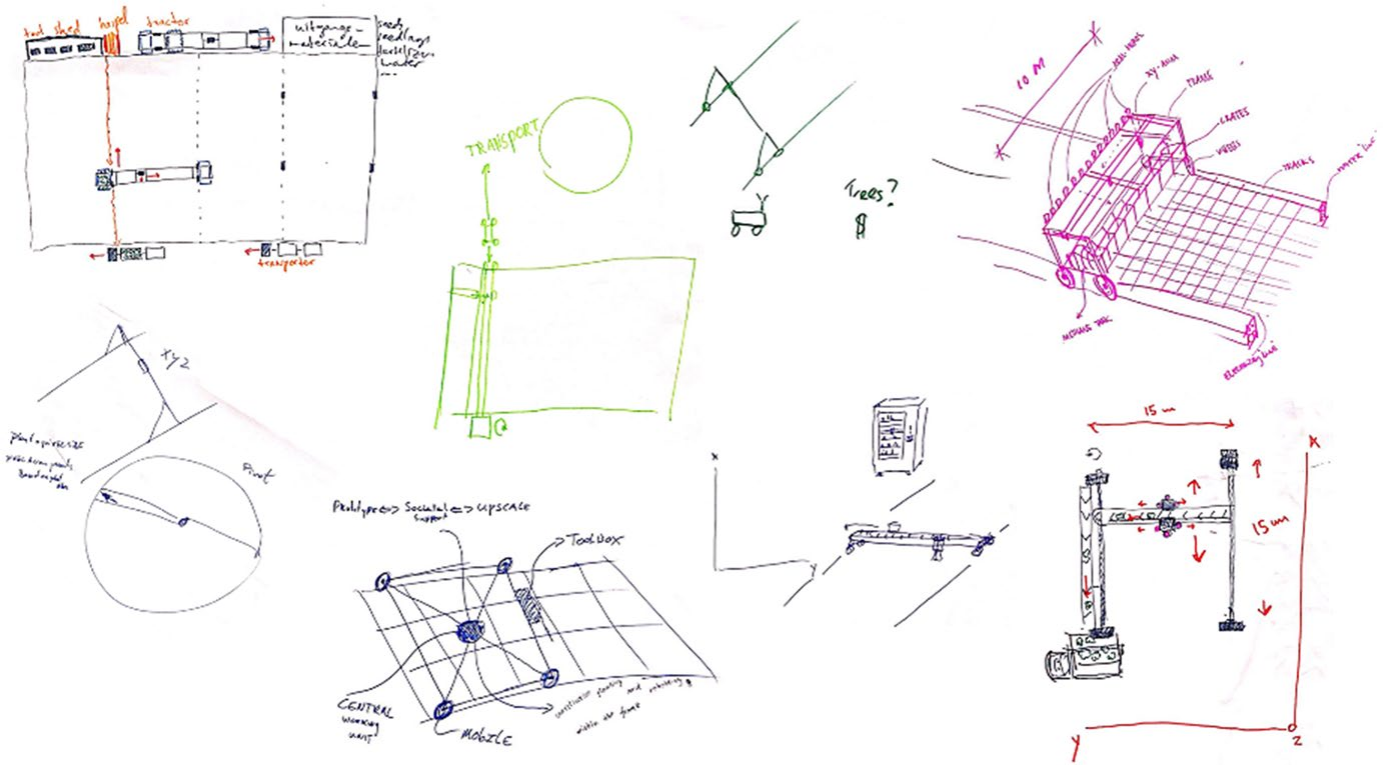
A variety of drawings produced by participants at the pixel cropping robotics workshop at Wageningen in 2019, all approaching automation through similar forms

The World Café workshop participants and student teams moved next towards the ideation phase in which functions were integrated into comprehensive designs. In each setting, we asked for visual renderings of the imagined tools. At the workshop, participants used flipcharts to sketch out their ideas while drawing on the lists of functions and solutions developed in earlier phases of the World Café. A striking outcome of this session was that nearly all integrated designs appeared highly similar in form: most drew robots composed of a gantry frame carrying tools over a field using Cartesian navigation (Fig. 4). Among these designs there was some variation between groups in how they addressed the different functions of the robot, for example whether the tool rolled independently on wheels or was mounted on fixed rails, but all were described as incorporating multiple functions to automate production from seeding to harvest.

**Fig. 5:**
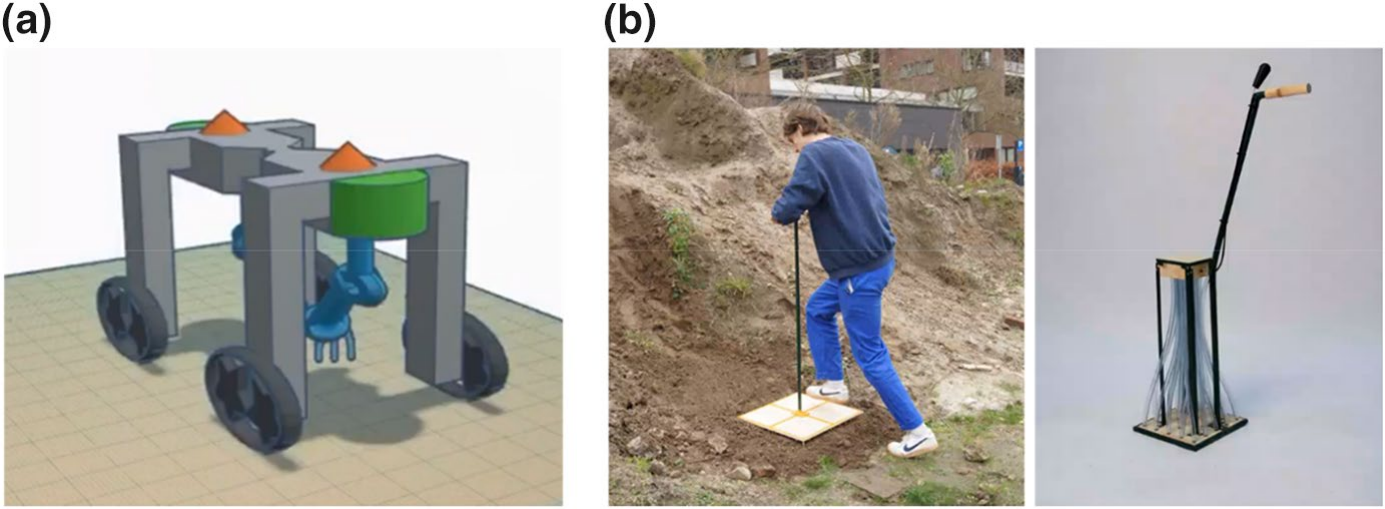
Mock-up for the “high-tech” robot model designed to seed and weed cereals in pixel plots, created by the WUR student consultancy project team, 2020 (**a**); prototypes for a two-piece analogue seeding tool developed by DAE students for sowing cereals in 50 cm × 50 cm pixels (**b**) (Objects and images in panel **b** made by Mick Thörig and Floris Meijer, 2019)

Students in the consultancy team tackled the integration phase by selecting options from their morphological charts and combining them to create two robot design sketches. For one version, which they called the “high-tech” model, they selected the most state-of-the-art options for each function (Fig. 5a). The consultancy team’s second model was designed to be more economically feasible; for this model they selected tools already commonly in use (e.g., a diesel engine instead of solar power, a hoe weeder instead of electrothermal weeding arms). The two models were presented as representative of a spectrum of options book-ended by a lower-tech but currently feasible and economically accessible tool versus a more comprehensive tool with less established and more expensive technology but greater autonomy. The design school team took an entirely different direction, abandoning the idea of a robot altogether and instead designing analogue hand-tools. Drawing on their interview with the field technician and their observations of her movements, the design school students came to the conclusion that a simple hand-tool that could be used immediately would have a greater impact on lessening her burden of manual labor than aspirational robotic equipment. They developed a simple, two-part seeding tool and fabricated working prototypes; the tools were light weight, required no power source, had few moving parts, were simple to operate, and were ergonomically designed so a farmer could use them while standing (Fig. 5b).

### Shifting Priorities on the Farm

At the Lochem site we observed various stages of developing a pilot commercial pixel farm, speaking with the farmer at key moments in the planning, execution, and reflection of the first growing season, including practical trials with a pixel cropping robot prototype. Throughout this process we witnessed several pivotal moments during which the farmer shifted or re-formulated his approach as new challenges and unexpected outcomes presented themselves.

At the start of the season, the farmer’s plan was to use a small tractor to manage the soil preparation, sowing, and planting in the pixel plot. On a visit to the farm during the growing season, we observed emerging management challenges related to these mechanization ambitions. After marking out the pixel field with the tractor, the farmer had become concerned about soil compaction and changed plans: “We started to do it with a planting machine but you had to drive through a [whole] row for maybe one or two pixels…and I didn’t want to go drive the tractor all the way through the land every time.” Abandoning the tractor meant doing the planting manually: “…we had to do it by hand. Every single pixel. And there was about 4000 in the hectare. I’d say a month at least it took us to get all the plants in.” The farmer deemed this an unacceptable time and labor burden and indicated he would not do it this way again in the future, later reflecting, “it was a hard time.”

The farmer also intended to use a robot prototype to weed the field having arranged for a robotics developer to use his field as a testing site in exchange for weeding services. Yet the robot was not fully functional at the time when weeding became necessary, so the farmer and his team started hand weeding. Following a period of particularly hot and dry weather the farmer observed that the seedlings in the weeded pixels fared worse than those in the un-weeded pixels: “We cleaned a few pixels during the warm days, but the crops, those ones died. Too warm, the ground dried out.” Following this observation, he decided to discontinue the hand-weeding efforts. No further weeding was done during the growing season, except for small test areas when the robot eventually arrived.

**Fig. 6:**
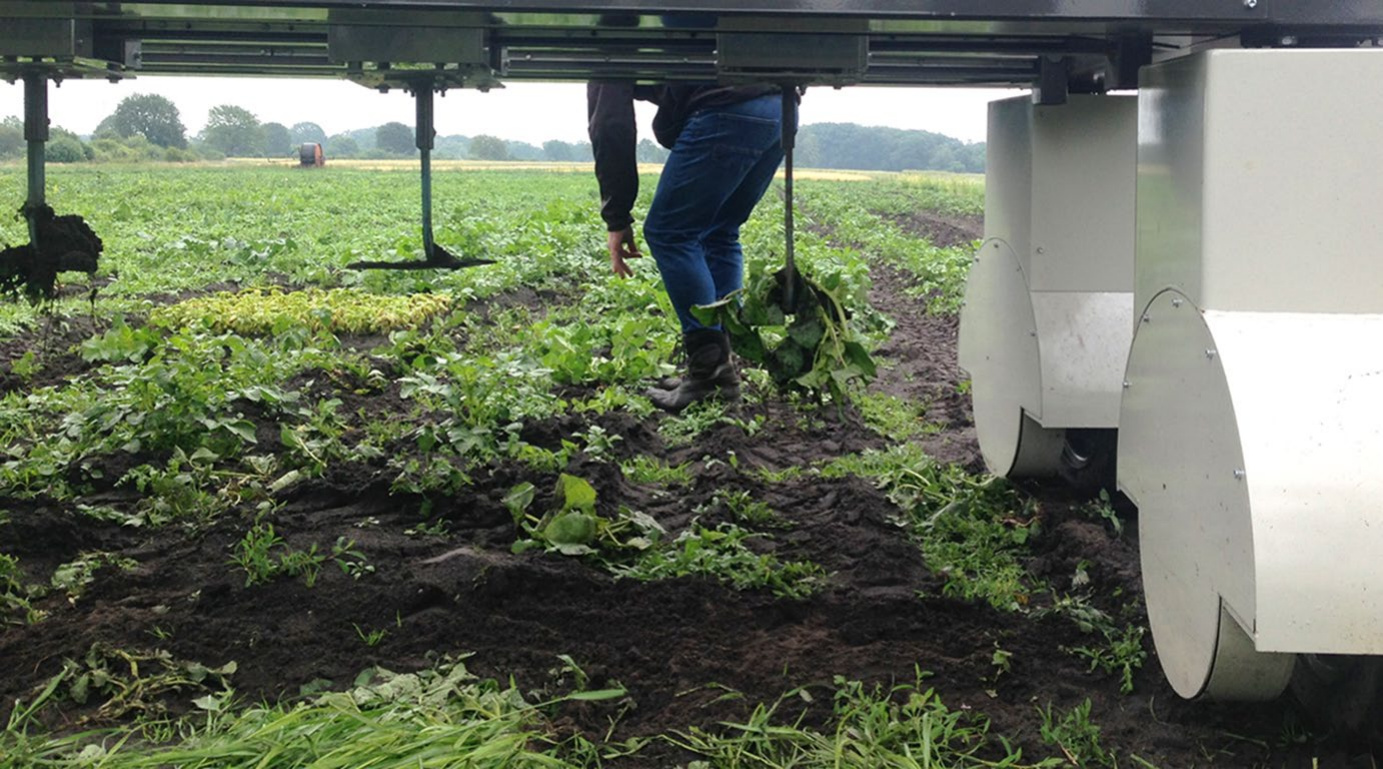
The Lochem farmer walks behind a prototype weeding robot and pulls out the weeds it missed, summer 2020

We next visited the Lochem farm to watch one of the robot trials, during which we saw the farmer walking behind the robot as it weeded a row of pixels (Fig. 6). In part he appeared to be observing how the machine operated, but he was also cleaning up after the robot. The robot was imprecise and missed weeds periodically, which the farmer then pulled himself. This form of monitoring the robot, or even collaborating with it, was a middle ground we had not seen explored in the other design projects and discussions. In an interview a few weeks after the robot’s weeding demonstration, the farmer reflected that developing the weeding function of the robot prototype was in his opinion no longer a priority stating, “I’m slightly changed now, the way I see crops are growing now, I’m not too bothered with weeds anymore.” The farmer expressed that he felt the assistance of the robot should be instead directed towards alleviating the more essential and time-consuming task of planting crops in the field.

During a mid-season interview we discussed the broader implications of automation. Asking whether the farmer saw the robot as a threat to his livelihood or to his approach as a self-identified agroecological farmer, he replied that he would welcome a tool that would help to alleviate the monotonous farming tasks that required a lot of time but little intellectual input. With the freed up time, the farmer reasoned, he would be able to devote more energy to walking through his fields, an activity which he both enjoys and which helps him to learn about his farming system and make more informed management decisions: “I’d rather be in the fields and look at crops and decide what to do instead of sitting on the tractor…looking at plants, touching plants, taking out a plant and looking at the roots…I walk through the field now and I’m really happy.” In this way, we understood that the automation of mundane tasks would allow him to deepen his connection to his farm and the natural ecosystem within which it is embedded and would not occasion a change in his identity as an agroecological farmer. This rationale echoed closely what some of the farming systems researchers had discussed previously regarding a desire to both foster connection and to open up time for thinking, which the Lochem farmer indicated could both be enhanced—not diminished—by the assistance of automation.

After the growing season had concluded, we again met with the farmer to discuss his reflections on the trial. During this conversation we focused on his shifting perspectives regarding the value of weeding and the tasks he would like to see a robot take over. Here, he stuck to his conviction that weeding was no longer necessary and emphasized his desire to instead have a planting robot. Additionally, the farmer had conducted a post-season survey with customers who had rented pixels in a community supported agriculture (CSA) scheme during the 2020 season. Many of the CSA members had indicated that they wished they had had more opportunities to interact with the pixel farm and participate in cultivation activities. Thus, in addition to asking the robot developers to focus on planting and sowing, the farmer had decided to try hosting a series of open days at the start of the next growing season during which the CSA members could plant their own pixels. The farmer anticipated that the labor reduction afforded by the combined efforts of the CSA members and the new robot functions would make the field preparation manageable enough to engage in a second pixel cropping trial season.

## Discussion: Beyond the Dream of Total Automation

Through a series of exploratory research inquiries, we asked people from different backgrounds to imagine automated tools for pixel cropping, using pixel cropping as a particular translation of agroecology and a stand in for a broader range of diversified alternatives to monocultures in industrialized contexts. While we had previously identified the predominating approaches towards automation in open-field farming systems—each leading to reinforcement of a monocultural paradigm, with total replacement of humans by machines as a central goal—what arose in these happenings was a wider range of possible approaches and imagined relations between humans and robots. The spectrum of possible directions in which to take robotics that came out for pixel cropping highlighted different aspects of a set of interconnected dialogues: on the ideals and practices of agroecology, the socio-political concerns of mechanization, and how to approach design processes for sustainable agricultural transitions. Each of these suggest different ways of understanding and imagining automation for agroecological farming systems.

### Can an Ethos be Automated?

In the robotics engineering literature, the operational management of farming systems is described as an iterative loop which involves an actor (farmer or machine) receiving information, processing that information, deciding how to take responsive action, and then actuating the task. It has been considered that there are four levels to which parts of this loop can be automated, book-ended by full human labor and total automation (van Mourik et al., 2021). What we saw come out of the World Café workshop, and to some extent also the consultancy project, were robot designs that largely conformed to the mainstream aspiration of total automation as well as the homogeneity of form generally adhered to in robotic equipment coming out of precision farming programs. In both settings, participants envisioned tools which were meant to fully replace human hands in the execution of a predefined list of tasks, providing evidence that the dream of total automation exists not only in the monocultural paradigm, but also in the minds of those confronted with a polyculture. Despite staying within a limited range of robot forms, a key deviation from dominant automation narratives was evident in how participants in our happenings imagined the information processing and decision making stages within the operational management loop.

Through our happenings we learned that to create a fully automated pixel cropping robot implies not only the development of a wider range of plant recognition and actuation functions (e.g., sowing, weeding, and harvesting multiple different crops^7^), but also programming into a robot the ethos of agroecological farming which draws on a multiplicity of ways of knowing soils, plants, and other (un)invited flora and fauna, their functions, and interactions (Tittonell et al., 2020). Bringing agroecological ways of knowing and responding to plants into the robotic imagination opens space for new thinking about how crops are related to and interacted with in the field—whether by humans or by machines, or newly imagined combinations of the two—that has not yet entered the mainstream monoculture paradigm. In monocultures, where crops are approached as passive objects awaiting manipulation, receiving information, processing it, and deciding how to act is a mono-linear process which leads to a straight-forward management action. In a diversified cropping system like a pixel farm, which leverages ecological controls rather than external inputs, distinctions inherent in the linear input—output and binary crop/weed monocultural approach become blurry and often irrelevant. The system itself becomes an active participant in the farming activity as interactions such as those between crops and insects emerge and self-regulate (Tsing, 2015). As such, the stages of the operational management loop become much more complex, the decision making fuzzier, and the range of possible actions more extensive. To accommodate this fuzziness, design might need to be conceived not as leading to a particular robotic device with functionalities which match tasks predefined in an operational management loop, but as part of a process that reconfigures and rethinks relations while maintaining an ongoing openness to adapt and learn from these relations.

Following Escobar (2018, p. x), we can think of design as ontological: bringing about particular ways of being, doing, and knowing. Fully automating agroecological practices would imply that robotics developers would have to make choices about what the essential interactions are within the system (e.g., pest—natural enemy) that should be optimized or facilitated, and then translate this knowledge into articulated actions supported by the technology. If the final phase of automation is to be understood as human-free, the robot would have to be equipped with the ability to learn and make these decisions in real time, a prospect which triggers new questions about what ontologies robots should be taught to adopt (Legun & Burch, 2021), and the ethical implications of these choices (Ryan et al., 2021; van der Burg et al., 2019).

In a fully automated scenario, success presumes that the human manager only relates to the robot and the data it generates and not to the farm field directly, calling into question what trajectories for farming knowledge and farmer identities such tools may perpetuate or precipitate (Carolan, 2020; Rotz et al., 2019). A recent news story explained that farmers who had acquired a weeding robot could drop the robot off at the field and then happily sit back and watch television while the machine did the work for them (Radersma, 2020). As this story suggests, the type of tacit, sensuous knowledge a farmer gains by spending time in the field risks being replaced by data-mediated knowledge when farming operations are automated (Carolan, 2020; Kuch et al., 2020). We heard this concern raised by participants in the agroecology discussion group. Yet, the Lochem farmer later provided an alternative and more nuanced view in which the desirable sensory experience of farming could be separated from the drudgery of doing monotonous tasks. This distinction offers an interesting new way to think about the space automation might occupy—and open up—in the agroecological farm operations management cycle that would not further disconnect a farmer from their fields but instead afford the time to engage other forms of knowledge and care (Puig de la Bellacasa, 2015; Smith & Fressoli, 2021). Being a farmer who uses robots could mean a shift in how farmers self-identify or are identified by peers (Burton, 2004), but the Lochem farmer’s story suggests it does not have to mean a shift from being a tractor driver to being a computer controller (or TV watcher). Engaging in the forms of robot-enabled care the Lochem farmer described could also become associated with what it means to be a ‘good’ agroecological farmer in a post-productivist paradigm (Burton, 2004).

### Working with Robots, or Going Without

The outcomes of our pixel cropping inquiry suggest that there may be room for achieving novel aims within the bounds of dominant automation narratives. We saw evidence that the types of relations between farmers, ecologies, and communities that “technoscientific futurity” (Puig de la Bellacasa, 2015) might occasion could be beneficial and not necessarily contrary to agroecological care, as unfolded in Lochem. There, we saw the farmer envision a type of agroecological connection that is both enabled by automation and that allows for cultivating an ethos—one that values production but at the same time allows for other ways of relating to crops, soils, ecologies, and the wider community. This vision transcends the opposition highlighted by other scholars in which care oriented around vital practices and experiences is considered to be in danger of being “discounted, or crushed, by the productionist ethos” (Puig de la Bellacasa, 2015, p. 708). Additionally, centering crop husbandry as an act of managing ecological relations provides a profoundly different way of thinking about farming—and enabling technologies—within large-scale production environments (Sukkel, 2020). These outcomes recall other work suggesting that maintaining some of the tested tropes of monocultural farming may provide a conceptual and operational bridge for farmers reassembling their systems towards sustainability goals (e.g. strip cropping, Ditzler et al. 2021) or technology transitions (e.g. "robot-ready" apple orchards, Legun & Burch, 2021).

We also observed the limits imposed by monocultural thinking and saw evidence that a loosening of definitions may be needed in the case of automating agroecology. First, broadening the notion of automation could allow room for blended models of engagement that may be more locally appropriate than full robotic control (and potentially more accommodating to safety regulations, see Lowenberg‐DeBoer et al*.* (2021)). When the Lochem farmer walked behind the weeding robot picking out weeds it had missed, he demonstrated a human—robot collaboration, what could be viewed as a ‘co-bot’ scenario, that goes beyond the “supervised control” described by van Mourik et al. (2021). This type of collaboration has given rise to a number of debates regarding the role of technologies as replacers or collaborators for humans (Ryan et al., 2021), and philosophical questions of who is adapting to or assisting who (Bissell, 2021). Smith and Fressoli (2021) provide a useful conceptual framework for thinking about “post-automation,” where encouraging a plurality of engagements with technology could provide an alternative to an essentialized future for automation. Human—robot collaboration however, is not well explored in the agricultural robotics literature (Vasconez et al., 2019). Rather, the farmer’s position is more commonly envisaged as described by Lowenberg‐DeBoer et al*.* (2021, p. 11) as sitting “in a vehicle at the edge of the field working on a computer”. Second, expanding the view of what is considered a “radical redesign” (Altieri et al., 2017; Hill & MacRae, 1996; Pissonnier et al., 2019) could provide necessary room for using familiar or ‘old’ tools in new ways (Stuiver, 2006; van der Veen, 2010). In different contexts the radical option might emerge in an unpopulated middle ground: as an opportunity to engage more deeply with community (as on the Lochem farm), as a change of practices mid-season (as the Lochem farmer did with weeding), as embracing the ‘low-tech’ (as the design school students did), or as commons-based peer production (Smith & Fressoli, 2021) exemplified in projects like the French collective L’Atelier Paysan (Salembier et al., 2020), the “Slow Tools” movement in the United States, and the international open-source exchange platform Farm Hack.

The variety of appropriate ways in which we saw automation could be used for agroecological aims reflects the locally embedded nature of agroecology, and its emphasis on diversity—not only of crops but also of system actors. In our happenings, we witnessed a range of tool designs emerge from the same context but devised by different types of practitioners, highlighting the potential for the structure and content of design processes to steer outcomes towards different visions for automation. A diversity of design processes and designers is therefore likely essential to address the multifaceted design needs of automated farming futures, needs which will vary in relation to the context-specific political ecologies that automation may precipitate. Locally-adapted and diverse design processes could help to avoid that the limited values and norms of homogeneous designers dictate a perpetuation of business-as-usual (Bronson, 2019; Escobar, 2018), that valued human or non-human actors are displaced (Schmitz & Moss, 2015), or that one-way technology development fuels a trajectory towards a robot-managed mega-monoculture dystopia (Daum, 2021).

Our findings also suggest that an iterative and reflexive design—test—learn—redesign process, as is commonly used in farming systems design and innovation approaches (e.g. Rossing et al., 2021) is relevant not just for the design of production systems, farms, or landscapes, but also for the farming implements themselves (Rose et al., 2021). On the Lochem farm, the prospect of using a robot to facilitate pixel cropping had initially led the farmer to experiment with a novel and risky form of farming, yet as the season progressed, we observed that the interplay between the not yet fully realized robot and emerging complexity of the cropping system in turn led to surprising lessons about not needing certain presumed robotic functions. This example highlights the importance of making room for what Meynard et al. (2017) and Salembier et al. (2020) have called “coupled innovations”, in which the design process is shifted from aiming to achieve a singular end goal (how to automate system *x*?) and rather towards a feedback process driven by the underlying ethos of the desired system (how to facilitate the processes and outcomes we want?) for which the right implement may or may not be a robot, or might involve combinations of humans, manual tools, and forms of automation.

## Conclusions

This paper raises questions on how to realize automation within agroecological cropping systems, given that the predominating directions for automation playing out in the open-field agricultural sphere are aimed not at amplifying diversified cropping systems but at enabling the industrial monocultural paradigm to persist in the face of shifting societal demands and economic logics. Through the case of pixel cropping, we explored what might come out when various actors considered automation and designed tools specifically for an agroecological paradigm in which complexity is embraced, ecological cycles are fostered, and the boundaries between binaries such as crop/weed and labor/fulfillment are blurred. What emerged was a diversity of approaches and imaginations for automation, which ranged from full automation, to collaborative modes, to fully analogue tools. From these examples we learn that automating agroecology will require the same situated and diverse range of approaches and actors that is at the foundation of the agroecology ethos, and therefore a rethinking of what automation might mean in different contexts.

Drawing on the findings of the pixel cropping case, we propose to engage with the notion of automation for agroecology as a dynamic range of context-dependent options and directions, rather than an all-or-nothing binary. We posit that this will be more realistic regarding technical feasibility, more accommodating to the ethos of agroecology, and more malleable to the diversity of socio-political contexts within which new farming technologies will land. In the design of (partially) automated tools, expanding the notion of automation would require envisioning design not as a linear development through which an object is created following a preconceived blueprint of tasks to be relieved from human hands. Rather, design could be conceived as a place-based, iterative, and dynamic feedback process involving designers, researchers, farmers and farm workers, non-human system residents, and other locally relevant groups, thereby creating space for (radical) middle ways to emerge. For researchers and designers, we posit that such a process should start with seeking to understand both the ecological and human characteristics of the agroecological system being designed for, rather than starting with monocultural assumptions and seeking to fit them onto diverse cropping systems. Integrating the diverse knowledges and embodied practices of agroecological farmers and farm workers will be key for re-orienting automation away from the constraints of monocultures, and potentially towards more technically achievable applications that can respond to dynamic conditions and incorporate ongoing learning processes. For practitioners and researchers of agroecology wary of automation, a more expansive view of what automation could mean in different circumstances might allow new labor solutions to emerge, in conjunction with new roles and experiences for practitioners and their communities, potentially creating opportunities for wider uptake of agroecological modes of crop care.

## Footnotes

1 We tentatively define *robot*s as “perceptive programmable machines”, following Bechar and Vigneault (2017).

2 We use the term ‘open-field farming’ to refer to cropping systems in which plants (arable and vegetable crops) are grown outdoors in soil, as opposed to in controlled growth environments such as greenhouses.

3 For instance, the time required to cultivate a maize crop (from soil preparation to harvest) mechanically is approximately 6–10 h per hectare (WUR, 2009), compared to an estimate of more than 2000 h per hectare when done by hand in a less mechanized context (Ditzler et al., 2019).

4 The same pattern has been projected to hold for small-scale autonomous field equipment, particularly in places where safety regulations favor larger farm operations (Lowenberg-DeBoer et al., 2021).

5 For an overview (not necessarily complete) of open-field farming robots in development and on the market at the time of writing, see: https://misset.com/field-robots/field-robots/.

6 We use the term *ethos* following van Dooren and Rose (2016) to mean not only a particular way of defining the elements, objects, and subjects the world is made up of, but also as a mood, an aesthetic, a way of narrating, and a collectively shared attitude.

7 Existing research projects have channeled entire multi-year grants towards the development of tools that can identify and remove a single species of weed from between a single species of crop planted in monocultural and row-based field layouts (e.g., Nieuwenhuizen et al., 2010). To move towards a system in which a robot could encounter any plant, growing anywhere, and then determine based on its identity whether or how to engage with it, would require advanced applications of visioning, sensing, and machine learning not yet achieved.
